# Case report: Intensive rehabilitation program delivered before and after single-event multilevel surgery in a girl with diplegic cerebral palsy

**DOI:** 10.3389/fneur.2023.1323697

**Published:** 2024-01-12

**Authors:** Alessandra Crecchi, Alessandra Tozzini, Roberta Benedetti, Marco Maltinti, Luca Bonfiglio

**Affiliations:** ^1^Unit of Developmental Neurorehabilitation, Maternal and Child Department, Pisa University Hospital, Pisa, Italy; ^2^Department of Translational Research on New Technology in Medicine and Surgery, University of Pisa, Pisa, Italy; ^3^1st Unit of Orthopaedics and Trauma, Pisa University Hospital, Pisa, Italy

**Keywords:** diplegic cerebral palsy, gait disorders, tendon lengthening, gait analysis, neurocognitive rehabilitation

## Abstract

**Introduction:**

Diplegic cerebral palsy (CP) is often associated with musculoskeletal disorders that contribute to worsen walking function. The standard care in these cases is single-event multilevel surgery (SEMLS) followed by rehabilitation. Our aim was to investigate whether a rehabilitation program starting even before SEML could add a benefit with respect to standard postoperative programs considered by previous research.

**Methods:**

From 2 months before to 13 months after SEMLS (except for the first month after surgery), the participant underwent a motor training focused on ROM exercises with tactile and kinaesthetic feedback. Walking performance, walking capacity, and quality-of-life were assessed before and after SEMLS at different follow-up times.

**Results:**

Walking capacity improved 3 months after SEMLS (i.e., earlier than in current literature) and walking performance improved 12 months after SEMLS (instead of simply returning to baseline as previously reported), with a positive impact on quality-of-life.

**Conclusions:**

This case suggests that a rehabilitation program starting even before SEMLS could add benefits over walking function and quality-of-life of children with diplegic CP compared to postoperative programs only.

## 1 Introduction

Cerebral Palsy (CP) is defined as a group of disorders of motor control and posture development occurring as a result of a non-progressive impairment of the developing central nervous system (1). CP is a common cause of motor disabilities in children and, especially for bilateral forms, often has serious consequences on walking abilities. In addition, patients with CP often present a series of secondary musculoskeletal disorders that in turn affect walking function and may worsen with age, such as shortening of muscle-tendon units, torsional deformities of bones, and instability of joints (2). The diplegic form of CP is often combined with crouch gait, which is characterized by dorsiflexion of the ankle as well as knee and hip flexion during the stance phase of gait. This, besides interfering with daily life activities and social participation, could lead to the loss of walking ability in adult age (3, 4).

Orthopedic surgery procedures are often recommended to treat such musculoskeletal deformities: single-event multilevel surgery (SEMLS), defined as two or more soft-tissue or bony surgical procedures at two or more anatomical levels during one operative procedure-combined with post-operative rehabilitation- is considered the standard of care in walking children affected by spastic diplegia (5, 6).

SEMLS, by favoring alignment and lever arm function, results in a good improvement of walking capacity (i.e., what a child can do in a standardized controlled environment) detected 1 year after surgery and long-term persistent (5, 7, 8). However, less is known about the temporal evolution of walking performance (i.e., how a child actually moves around in his/her current environment in everyday life) and quality-of-life during the post-surgery rehabilitation period. These topics are scarcely taken into consideration in literature, although they are potentially able to condition the post-surgical outcome in terms of daily life activities and social participation. In addition, there is little information about what characteristics the rehabilitative intervention should have (in terms of type of exercises, sessions per week and duration of the whole program) in order to optimize SEMLS effectiveness. Furthermore, to the best of our knowledge, no study has so far reported the beginning of rehabilitative intervention prior to surgery.

In this paper, we report a case of a girl diagnosed with diplegic CP and crouch gait treated with SEMLS in our hospital. Its peculiarity lies in SEMLS not only being followed by the rehabilitative program, as usual, but also being preceded by it. By analogy with other surgical procedures involving lower limbs articular function (9, 10), this had the purpose of both preparing the patient to SEMLS and maximizing its results through both muscular strengthening and mobilizing exercises. We describe here patient outcomes in terms of gross motor function, walking capacity, walking performance, and quality-of-life.

## 2 Methods

### 2.1 Case description

We report here the case of a 14-year-old girl with CP, caused by premature birth and cerebral hypoxia due to uterine rupture at 27 weeks of gestation. Although since early childhood she had been enrolled in a regular low-intensity (two-three times a week) rehabilitation programme and treated with BoNT-A injections, she unfortunately developed crouch gait in adolescence with flexed and adducted hips, flexed and increasingly painful knees. Moreover, mild bilateral rotational deformities of long bones (femoral medial torsion and tibial lateral torsion) as well as a bilateral planovalgus foot with talonavicular joint dislocation (without forefoot abduction) completed the picture of a “lever arm disease”. For these reasons, she was referred for evaluation to the orthopedic surgeon of our hospital, who opted at this stage for soft-tissue SEMLS only, postponing the correction of skeletal deformities to a possible subsequent phase. She had never had relevant comorbidity, or functional surgery before.

Both patient and parents, as legal representatives, gave their written informed consent to publication. Ethical approval was obtained from the Pediatric Ethics Committee of the Tuscany Region (protocol number: CR_02/2018; date of approval: 16 October 2018).

### 2.2 Pre-operative rehabilitation

**Figure 1:**
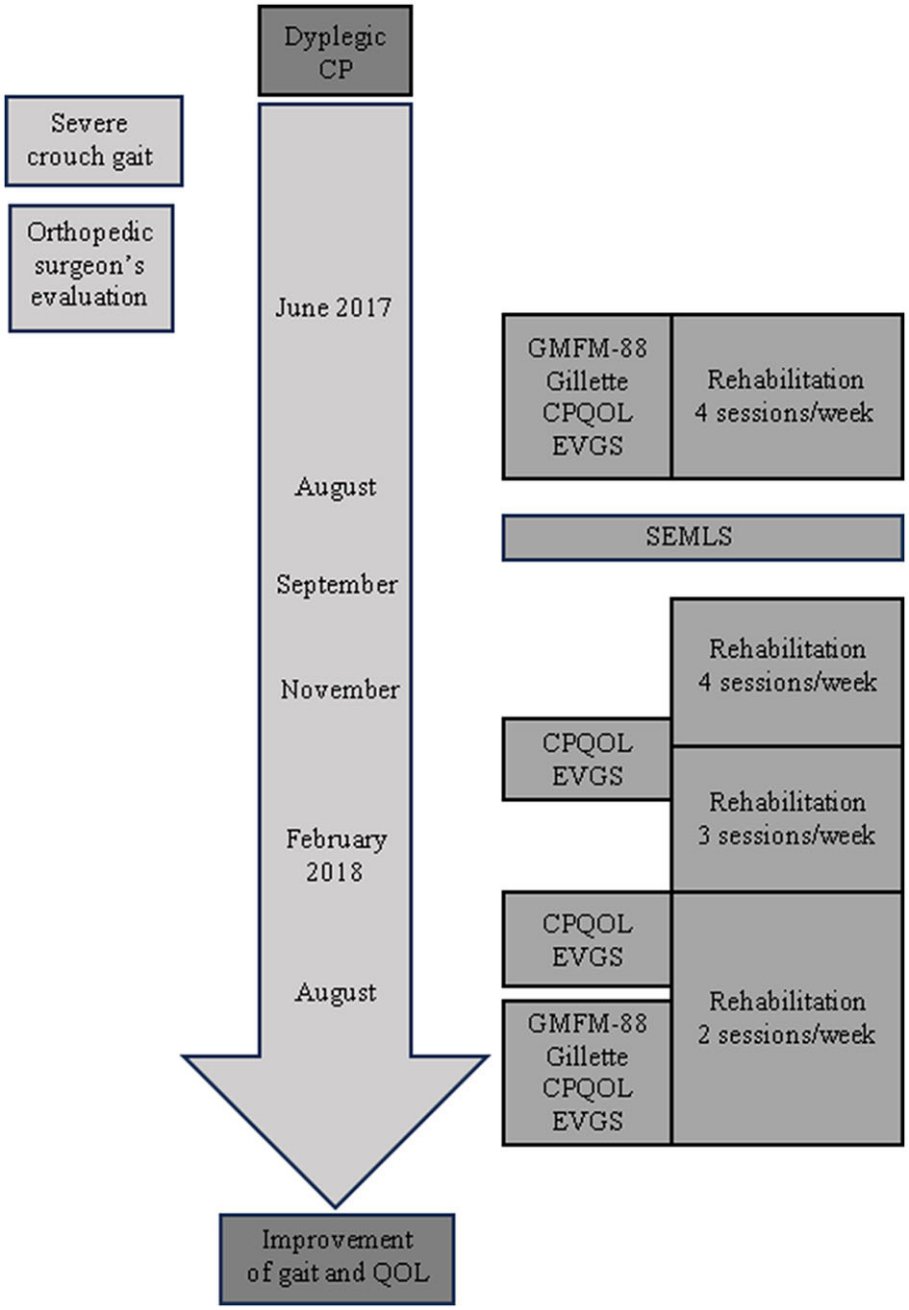
Case timeline.

As shown in the timeline (Figure 1), the patient began an intensive rehabilitation period for 2 months before SEMLS was performed, which consisted in 4 sessions per week (4 h/week) of supine stretching exercises along with supine and standing active-assisted range of motion (ROM) exercises for hips and knees with tactile and kinaesthetic feedback.

During active-assisted ROM exercises, the participant's limb was moved by the physiotherapist along the edges of a simple 2D geometric figure. While movement was performed with eyes closed, the participant had both to select and gather relevant tactile and kinaesthetic information in order to create a visual mental representation of the shape. That representation had to be encoded and subsequently retrieved from memory to be compared with a series of geometric figures to recognize the one corresponding to the performed movement. This type of exercise complied with the principles of the so-called “neurocognitive rehabilitative approach” (11).

### 2.3 Surgical intervention

The surgical procedure consisted of different successive stages within a single surgical session: (a) the origins of adductor muscles (i.e., adductor longus, adductor brevis, and adductor magnus) were cut transversely in order to partially resolve their contracture; (b) the tendon of the ileo-psoas muscle was lengthened by Z-plasty; (c) the tendons of both the semitendinosus muscle and the gracilis muscle were lengthened by Z-plasty, whereas the semimembranosus muscle was lengthened by transverse incision at two levels of tendinous fibers on its deep side; and, finally, (d) the biceps femoris tendon was lengthened by incising transversely at two levels its tendinous portion in order to leave the muscle's fibers intact. This procedure was performed in both legs, except for the biceps muscle lengthening that was performed only on the left leg. After surgery, the patient had plaster casts applied on her legs that were removed 1 month later; then, she wore rigid knee braces during the night for another 2 months.

### 2.4 Post-operative rehabilitation

One month after surgery, when plaster casts were removed, she started an intensive period of rehabilitation with 4 sessions per week (4 h/week) for 2 months, and, then, three sessions per week (3 h/week) for another 4 months, in which she performed supine stretching exercises together with supine and standing active-assisted ROM exercises for hips and knees with kinaesthetic and tactile feedback; finally, until 12 months after surgery, she performed standing weight transfer exercises with multisensorial (kinaesthetic, tactile, and visual) feedback for two sessions/week (2 h/week).

Active-assisted ROM exercises were carried out in the same way as in the pre-operative rehabilitation. Weight transfer exercises were performed either in neutral or stride position. In the former, the participant had to maintain tactile contact with sponges inserted between back and wall and to maintain visual contact with targets placed in front of her. In the latter, the assumption of feet position was assisted by the physiotherapist and, based on kinaesthetic information only (being feet screened), the participant had to recognize her feet reciprocal position by comparison with a series of depicted combinations placed in front of her. Also in this case, the sequence of cognitive operations that the participant had to perform, according to the “neurocognitive rehabilitative approach”, consisted in selecting and gathering relevant kinaesthetic information, creating and maintaining a mental representation of the current posture, and finally comparing it with a series of possible options to identify the actually taken posture (11).

### 2.5 Clinical assessment

The patient's clinical outcomes were assessed at different time-points, as can be seen from the timeline depicted in Figure 1. Both gross motor abilities and walking performance were assessed immediately before (baseline) and 12 months after SEMLS by means of the Gross Motor Function Measure (GMFM-88) and Gillette Functional Assessment Questionnaire (Gillette FAQ), respectively. In addition, walking capacity and overall quality-of-life were measured at baseline and 3, 6, and 12 months after SEMLS by means of the Edinburgh Visual Gait Score for Use in Cerebral Palsy (CP-EVGS) (8) and Cerebral Palsy Quality-of-Life Questionnaire (CP-QOL), respectively. The CP-EVGS was assigned through the observation of video recordings of gait as described by Read et al. (8).

## 3 Results and discussion

Table 1 shows scores for each clinical scale before and after SEMLS at different follow-up times.

**Table 1:** Scores of clinical scales before and after SEMLS.

**Clinical scales**	**Before**	**3 mos. after**	**6 mos. after**	**12 mos. after**	**Percentages of change**
GMFM-88	72 (III)	–	–	75 (III)	4%
CP-EVGS	29 (5)	26 (0)	22 (0)	18 (0)	37.9%
Gillette FAQ	6	–	–	8	33.3%
CP-QOL	297 (44)	319 (46)	328 (42)	392 (30)	32% (31.8%)

GMFM-88, the corresponding level is shown in brackets; CP-EVGS, values are expressed as the sum of scores of the two limbs; differential values between the two limbs are shown in brackets. CP-QOL, pain subscale scores are shown in brackets. Percentages of change express the final improvement at 12 months after SEMLS compared to baseline.

By keeping in mind these data, the following considerations can be made: (a) even though the GMFM-88 global score improved by 4% 12 months after SEMLS, the GMFM-88 level, as expected, did not change; (b) the CP-EVGS score improved after the first follow-up and in the succeeding ones, with an inter-trial increase of at least three points corresponding to the minimal important change (MIC) for CP-EVGS (8) and a final improvement of 37.9% with respect to baseline 12 months after surgery (moreover, the differential value between limbs, equal to 5 before surgery, was already zeroed since the first follow-up after SEMLS); (c) the Gillette FAQ score showed an increase of 2 points corresponding to the MIC for Gillette FAQ (12) with a final improvement of 33.3% 12 months post-surgery; (d) the CP-QOL score improved after the first follow-up and increased at subsequent ones up to 32% with respect to baseline 12 months after SEMLS (with a relative improvement of 31.8% in Pain Domain). Furthermore, before SEMLS the patient was only just able to walk for short distances with two large base quad canes and used a wheelchair to cover longer distances, whereas 6 months after SEMLS she was able to walk for longer distances with only one large base quad cane and 1 year after surgery she was able to walk with two walking sticks with narrow quadri-support rubber ferrules.

From a review of the current literature on the topic, walking capacity indices (i.e., gait analysis parameters) turn out to improve between 9 and 12 months after SEMLS (6, 7). Conversely, walking performance indices (i.e., Functional Mobility Scale, FMS, or Mobility Questionnaire47, MobQuest47) invariably worsen in the first postoperative period (i.e., after 2–3 months) (13, 14) to regain previous baseline values only afterwards (i.e., after 6–12 months) (13, 14) and improve (if anything) even later (i.e., after 24 months) (13). Therefore, walking performance recovery after SEMLS seems to be at least delayed with respect to walking capacity. By reasoning in terms of the WHO International Classification of Functioning, Disability and Health (IFC) domains (6, 12), this could happen because, over and beyond the correction of the “body structure” domain that takes place at the time of intervention and soon reverberates upon the “body function” domain through the removal of biomechanical constraints, the CNS has also to adapt to the new musculoskeletal trim through a re-learning process in order to improve sensorimotor performance and motor control. This reasonably requires longer to be brought to completion since it implies a plastic re-organization of neural networks and could be better reflected onto walking performance rather than walking capacity.

Conversely, in the present case report walking capacity indices (CP-EVGS) significantly improved compared to baseline since the first follow-up 3 months after SEMLS and further improved after 6 and 12 months (with significant steps not only with respect to baseline, but also to each previous follow-up). In addition, walking performance indices (Gillette FAQ) considerably improved at the 12-month follow-up, whilst in previous literature they were only able at this time-point to recover baseline (i.e., pre-operative) levels. Even though clinical evaluations we used were not the same of previous literature (representing a possible limitation of the present study), it is important to point out that CP-EVGS (i.e., observational gait analysis) scores are known to well-correlate with instrumented gait analysis indices (8) and that FMS as well as Gillette FAQ have been proven suitable for depicting changes in children's walking performance after interventions (12).

Furthermore, our patient also had a progressive and constant improvement of her quality-of-life (CP-QOL) which, in combination with the performance index, also gives us an idea of the improvement of her participation level peaking at 6–12 months after the intervention. This aspect has often been overlooked previously, even though an improvement in those areas is crucial for both children's and families' wellbeing (15).

Since the main distinctive element of our case compared to those described in literature is the beginning of the rehabilitative program before SEMLS, we believe that the better overall outcome obtained is attributable to the fact that our patient followed a 2-month intensive rehabilitation period prior to SEMLS. On the one hand, this improvement could be generically attributed to the muscular strengthening of lower limbs and to the maintenance of an acceptable muscle-tendon compliance allowing the musculoskeletal system to better face the intervention and the following immobility period (9, 10). On the other hand, however, we could put forward an alternative or complementary hypothesis. Indeed, we speculate that, focusing the pre-operative physiotherapy intervention on kinaesthetic and tactile feedback of motor tasks, it may have favored neuronal plasticity phenomena within the sensorimotor network, thereby fostering (i.e., speeding up) both processes of motor re-learning (16) and motor control (17). In this way, a trained sensorimotor network would have become more responsive and adjustable to changes in sensory feedback induced by both the new musculoskeletal trim and changing environmental demands of everyday life, thus improving control abilities on motor output. This could better explain our results in terms of walking performance.

## 4 Conclusions

The present results suggest that an intensive rehabilitation program beginning before SEMLS could favor a better recovery of walking performance compared to programs limited to the postoperative period only.

We believe that these results, even if obtained in a single clinical case, are encouraging enough to justify further case-control trials to obtain confirmation on a larger scale. Moreover, functional neuroimaging studies would also be required in order to investigate neural plasticity phenomena underlying functional changes due to both pre- and post-SEMLS rehabilitation, with the final aim of combining SEMLS with the best possible rehabilitation plan for optimizing patients' outcome.

## Data availability statement

The original contributions presented in the study are included in the article/supplementary material, further inquiries can be directed to the corresponding author.

## Ethics statement

The studies involving humans were approved by Comitato Etico Regionale della Toscana—Pediatrico. The studies were conducted in accordance with the local legislation and institutional requirements. Written informed consent for participation in this study was provided by the participants' legal guardians/next of kin. Written informed consent was obtained from the participants' legal guardians/next of kin for the publication of any potentially identifiable images or data included in this article.

## Author contributions

AC: Investigation, Writing – original draft, Writing – review & editing. AT: Investigation, Methodology, Writing – review & editing. RB: Investigation, Writing – review & editing. MM: Methodology, Writing – original draft. LB: Conceptualization, Supervision, Writing – original draft, Writing – review & editing.
